# Effects of Supplementing Selenium-Enriched *Cardamine violifolia* to Laying Hens on Egg Quality and Yolk Antioxidant Capacity during Storage at 4 °C and 25 °C

**DOI:** 10.3390/foods13050802

**Published:** 2024-03-05

**Authors:** Kun Qin, Xin Cong, Hui Wang, Mengke Yan, Xianfeng Xu, Mingkang Liu, Fulong Song, Dan Wang, Xiao Xu, Jiangchao Zhao, Shuiyuan Cheng, Yulan Liu, Huiling Zhu

**Affiliations:** 1 Hubei Key Laboratory of Animal Nutrition and Feed Science, School of Animal Science and Nutritional Engineering, Wuhan Polytechnic University, Wuhan 430023, China; OldQinY@163.com (K.Q.); 15152099370@163.com (H.W.); yanmengke_yan@163.com (M.Y.); xianfen971325@163.com (X.X.); liumingkang0422@163.com (M.L.); songfulong4363@163.com (F.S.); wangdan@whpu.edu.cn (D.W.); xuxiao@whpu.edu.cn (X.X.); yulanflower@126.com (Y.L.); 2 National R&D Center for Se-Rich Agricultural Products Processing, School of Modern Industry for Selenium Science and Engineering, Wuhan Polytechnic University, Wuhan 430023, China; congxinwhpu@whpu.edu.cn (X.C.); s_y_cheng@sina.com (S.C.); 3 Department of Animal Science, Division of Agriculture, University of Arkansas, Fayetteville, NC 72701, USA; jzhao77@uark.edu

**Keywords:** *Cardamine violifolia*, selenium, egg storage, egg quality, antioxidant

## Abstract

Oxidative stress occurs in the process of egg storage. Antioxidants as feed additives can enhance egg quality and extend the shelf life of eggs. Selenium-enriched *Cardamine violifolia* (SEC) has strongly antioxidant properties. The objective of this study was to assess the effects of dietary supplementation with SEC on egg quality and the yolk antioxidant capacity of eggs stored at 4 °C and 25 °C. Four hundred fifty 65-week-old, Roman hens that were similar in laying rate (90.79 ± 1.69%) and body weight (2.19 ± 0.23 kg) were divided into 5 groups. The birds were fed diets supplemented with 0 mg/kg selenium (Se) (CON), 0.3 mg/kg Se from sodium selenite (SS), 0.3 mg/kg Se from Se-enriched yeast (SEY), 0.3 mg/kg Se for selenium-enriched *Cardamine violifolia* (SEC) or 0.3 mg/kg Se from Se-enriched *Cardamine violifolia* and 0.3 mg/kg Se from Se-enriched yeast (SEC + SEY) for 8 weeks. The eggs were collected on the 8th week and were analyzed for egg quality and oxidative stability of yolk during storage at 4 °C or 25 °C for 0, 2, 4, or 6 weeks. Dietary SEC and SEC + SEY supplementation increased the Haugh unit (HU) and albumen foam stability in eggs stored at 4 °C and 25 °C (*p* < 0.05). SS and SEC supplementation increased the yolk index in eggs stored at 25 °C (*p* < 0.05). SEC or SEC + SEY slowed down an increase in albumen pH and gel firmness in eggs stored at 4 °C and 25 °C (*p* < 0.05). Moreover, SEC or SEC + SEY alleviated the increase in malonaldehyde (MDA), and the decrease in total antioxidant capacity (T-AOC) level and total superoxide dismutase (T-SOD) activity in yolks stored at 4 °C and 25 °C (*p* < 0.05). These results indicate that SEC mitigated egg quality loss and improved the antioxidant capacity of yolks during storage. SEC supplementation would be advantageous to extend the shelf life of eggs.

## 1. Introduction

Eggs contain high-quality protein, minerals, vitamins, fats, and bioactive compounds [1]. Eggs, as a low-cost and highly nutritional food for humans, are consumed worldwide. Given the continual growth in egg consumption, China’s egg production exceeded 30 million tons in 2022. Storing eggs is common practice in commercial poultry production. Egg storage provides flexibility to meet market demand [2]. However, eggs are perishable products. During storage, eggs rapidly undergo weight loss and quality declines, which causes significant economic losses to the poultry industry [3,4]. 

From the moment an egg is laid, the albumen and yolk begin to undergo physical and chemical changes, causing changes in freshness, flavor, and palatability [5]. The albumen’s pH changes, and the loss of water in the egg increases due to CO_2_ loss through the shell during the longer storage time. This leads to the decomposition of the ovomucin-lysozyme complex, albumen dilution, and water infiltration into the yolk sack, which debilitates the vitelline membrane [6,7]. Additionally, Ebeid et al. [8] reported that egg storage (14 vs. 4 d) impaired the antioxidant capacity of hatched chicks, indicating that oxidative stress occurred during egg storage. The oxidative stress causes morphological changes in the albumen and vitelline membrane, thereby affecting the egg’s quality [9]. The inhibition of oxidation processes prevents any decrease in egg albumen quality and vitelline membrane elasticity [10]. Antioxidants can inhibit or slow down the rates of lipid and protein peroxidation, thus maintaining egg quality and extending the shelf life of the egg [11].

In poultry production, the hen’s nutrition has an important influence on egg quality. Dietary supplementation with antioxidants can enhance egg quality and the antioxidant capacity of laying hens [12]. A previous study demonstrated that dietary antioxidants helped to reduce lipid oxidation in egg powder during storage [13]. 

Selenium (Se) has antioxidant properties and can protect an organism against the actions of free radicals [14]. It is common practice in the poultry industry to supplement the diet of laying hens with Se to maintain poultry health. Traditionally, the Se source that has been used is inorganic sodium selenite and organic Se such as Se-enriched yeast. Se can mitigation the decrease in Haugh units (HU) during egg storage. Organic Se has a better effect than inorganic Se [15]. Moreover, as people are increasingly concerned about food safety and health, organic Se has more social acceptance than inorganic Se. The previous study also has shown that Se supplementation in Japanese quail diets extended the eggs’ shelf life during a storage period by improving HU and decreasing yolk fat peroxidation [16]. 

*Cardamine violifolia,* which is found in Enshi, Hubei, China, has an excellent ability to accumulate environmental Se. The Se concentration in the leaves of Se-enriched *Cardamine violifolia* (SEC) is greater than 700 mg/kg (dry weight), with over 85% of the complete Se deposited in the form of organic Se, such as selenocysteine and methylselenocysteine [17]. Our previous study indicated that dietary SEC supplementation enhanced the growth performance and antioxidant capacity of broilers [18]. Few studies have been performed to estimate the effects of SEC supplementation in a hen’s diet on egg quality during egg storage. Therefore, we decided to evaluate the effects of dietary supplementation with SEC on egg quality and the yolk antioxidant capacity of eggs stored at 4 °C or 25 °C.

## 2. Materials and Methods

### 2.1. Experimental Birds and Diets

The experimental protocols (WPU202204006) were reviewed and approved by the Animal Care and Use Committee of Wuhan Polytechnic University. A total of four hundred and fifty 65-week-old Roman laying hens with similar laying rates (90.79 ± 1.69%) and body weight (2.19 ± 0.23 kg) were assigned to 1 of 5 groups with 6 replicates of 15 birds each. The birds were fed diets supplemented with 0 mg/kg Se (basal, CON), 0.3 mg/kg Se from sodium selenite (SS), 0.3 mg/kg Se from Se-enriched yeast (SEY), 0.3 mg/kg Se from Se-enriched *Cardamine violifolia* (SEC) or 0.3 mg/kg Se from Se-enriched *Cardamine violifolia* and 0.3 mg/kg Se from Se-enriched yeast (SEC + SEY) for 8 wk. The basal diet has been reported previously (Table 1) [19] and was formulated in accordance with the National Research Council standards [20] to meet the nutrient requirements of laying hens with the exception of Se. At the end of the 8-week feeding trial, 480 eggs were harvested. The eggs had no defects (cracks or breaks), and the weight of eggs was close to the average egg weight for each replicate. 

*Cardamine violifolia* belongs to the Brassicaceae family and was discovered in Enshi, Hubei, China. *Cardamine violifolia* is suitable for growing in selenium-rich soil. Due to its ability to accumulate Se, fertilizers containing sodium selenite were used during the *Cardamine violifolia* growth process. 

In this study, the Se-enriched *Cardamine violifolia* (1430 mg/kg total Se content) was provided by Enshi Se-Run Material Engineering Technology Co., Ltd., Enshi, China. The SS and SEY were purchased from Angel Yeast Co., Ltd., Yichang, China.

### 2.2. Experimental Design and Egg Storage

Four hundred and eighty eggs were selected and divided into 5 dietary treatments with 6 replicates. The eggs were stored at 4 °C or 25 °C for 0, 2, 4, or 6 wk. All eggs were placed with small end down. From each storage period and temperature condition, 2 eggs from each replicate were collected to measure egg quality and egg functional parameters such as albumen firmness, albumen foam characteristics. The egg yolks were separated, collected and stored at −80 °C to detect their antioxidant capacity.

### 2.3. Egg Quality

Egg weight, albumen height, HU, and yolk color was analyzed using the Egg Analyzer (EA-01, Orka Technology Ltd., Ramat Hasharon, Israel). The weight loss of an egg during storage was calculated using the following equation: weight loss (%) = [(initial weight − final weight)/initial weight] × 100. Air chamber height was measured using electronic digital calipers (SH 1400025, Shanghai, China). Eggs were broken onto a flat surface, where the diameter and height of yolk were determined with electronic digital calipers. Yolk index was calculated as yolk height/yolk diameter, according to Sharp and Powell [21]. 

### 2.4. PH Measurement 

After the isolation of the yolk and albumen, they were poured into different beakers and homogenized. Then, the pH of the egg yolk or albumen was measured with a standard pH-meter (SevenExcellence S400-Basic, Mettler Toledo, Zurich, Switzerland). 

### 2.5. Albumen Foaming Capacity and Stability

Albumen foaming capacity and stability were determined according to the method described by Sauter and Montoure [22]. The albumen was poured into a beaker, mixed with a vortex mixer (WH-3, Shanghai Huxi Analysis Instrument Factory Co., Ltd., Shanghai, China) and subsequently whipped using a magnetic stirrer (Hei-Mix S, Heidolph Instruments GmbH & Co. KG, Schwabach, Germany) at 1400 rpm/min for 60 s. The blended albumen was then drained into 100 mL graduated cylinders. The foaming capacity was expressed as an increase in volume (%). 

After measuring the volume of albumen foam, foaming stability was determined by monitoring the foam for 30 min, then measuring the volume of albumen liquefaction. The foaming stability was calculated as follows:

foaming stability (%) = [(V1 − V2)/V1] × 100

V1—volume of the initial liquid phase (mL), V2—volume of albumen liquefaction (mL).

### 2.6. Antioxidant Indices

The total antioxidant capacity (T-AOC), the activity of total superoxide dismutase (T-SOD) and the content of malonaldehyde (MDA) in egg yolk were measured with a commercial assay kit (Nanjing Jiancheng institute of Bioengineering, Nanjing, China) according to the manufacturer’s instructions. Briefly, yolk samples (0.5 g) were homogenized in 2.0 mL PBS for the measurement of T-AOC and T-SOD and in 4.5 mL ethanol for the measurement of MDA. Then, the supernatants were collected after centrifugation (3000 rpm for 10 min at 4 °C). The protein concentration in the supernatants was measured using a Total Protein Quantitative Assay kit (Nanjing Jiancheng institute of Bioengineering, Nanjing, China). The T-AOC was detected according to the 2,2′-azinobis-(2-ethylbenzothiazoline-6-sulfonate) diammonium salt (ABTS) method with some modifications [23]. The supernatant was mixed with ABTS, while double-distilled water (which replaced the sample) was mixed with ABTS. All samples were incubated at 25 °C for 6 min. Different concentrations of 6-hydroxy-2,5,7,8-tetramethylchroman-2-carboxylic acid (Trolox) (0.1, 0.2, 0.4, 0.8, 1.0 mmol) were used to establish a standard curve. The absorbance of all samples was measured at 405 nm. According to the standard curve, the level of T-AOC was calculated. The T-AOC results were expressed as Trolox equivalent antioxidant capacity, which is defined as millimole Trolox/gram protein of egg yolk (mmol/g prot). T-SOD activity was analyzed using the hydroxylamine method. The supernatant was mixed with a reaction mixture containing xanthine, xanthine oxidase, and hydroxylamine hydrochloride, and incubated at 37 °C for 40 min. Then the developer was added, and the absorbance was measured at 550 nm. One unit of SOD is defined as the amount of the sample that causes an inhibition of 50% under the assay conditions. T-SOD activity was expressed as units/milligram protein for egg yolk (U/mg prot). To determine the MDA concentration, the supernatant was treated with thiobarbituric acid and boiled for 40 min to produce a colored product. Thereafter, the samples were cooled, added to n-butanol, and vortexed for 60 s. The mixture was centrifuged at 5000 rpm for 10 min. The upper layer was separated, and the absorbance was measured at 532 nm. The MDA concentration was expressed as nanomole/milligram protein of egg yolk (nmol/mg prot).

### 2.7. Statistical Analysis

Statistical procedures were conducted using SPSS17.0 statistical software (SPSS Inc., Chicago, IL, USA). Data were analyzed using a repeated measures approach in a general linear model procedure. Statistical modeling included the effects of dietary treatment, storage time, and interactions (dietary treatment by storage periods). Post hoc testing was performed using Duncan’s multiple comparison tests when a significant interaction was detected. The data were expressed as means and SEM. Differences were regarded as significant at *p* < 0.05 and tendencies were noted at 0.05 < *p* < 0.10.

## 3. Results

### 3.1. Weight Loss, Air Chamber Height, Albumen Height, HU and PH of Eggs Stored at 4 °C

The effects of different Se sources and storage time on weight loss, air chamber height, albumen height, albumen pH, and HU in eggs stored at 4 °C are presented in Table 2. Weight loss, air chamber height, and the albumen pH of the eggs significantly increased, but the albumen height and HU significantly decreased as storage time increased (*p* < 0.001). Dietary Se sources had no influence on weight loss, air chamber height, or the albumen pH of the eggs (*p* > 0.05). However, dietary Se supplementation alleviated the decrease of albumen height and HU compared to the CON group in eggs stored at 4 °C (*p* < 0.05). There was a dietary treatment × storage time interaction observed for albumen pH (*p* < 0.05). The albumen pH from the SS group was higher than that in the CON group in eggs stored at 4 °C for 6 wk (*p* < 0.05).

### 3.2. Yolk Color, Yolk Index and Yolk PH of Eggs Stored at 4 °C

As storage time increased, the yolk color and yolk pH of eggs significantly increased, whereas yolk index decreased (*p* < 0.001, Table 3). Dietary supplementation different Se sources did not affect yolk color, yolk index, or yolk pH (*p* > 0.05). There was no interaction between dietary treatment and storage time on yolk color, yolk index, or yolk pH (*p* > 0.05).

### 3.3. Albumen Foaming Capacity, Albumen Foam Stability, and Albumen Gel Firmness of Eggs Stored at 4 °C

Over the storage period, the albumen foaming capacity and gel firmness increased gradually, but foam stability decreased (*p* < 0.001, Table 4). The source of the Se used in dietary supplementation had no influence on the albumen foaming capacity, foam stability, and gel firmness of eggs storage at 4 °C (*p* > 0.05). There was a significant interaction between dietary treatment and storage time on albumen foam stability and gel firmness (*p* < 0.05). The foam stability in the SEY, SEC, and SEC + SEY group was higher than that in the CON group in eggs stored at 4 °C for 4 wk (*p* < 0.05). Moreover, dietary SS, SEY, and SEC + SEY supplementation mitigated the increase of albumen gel firmness compared to the CON group in eggs stored for 6 wk (*p* < 0.05).

### 3.4. Weight Loss, Air Chamber Height, Albumen Height, HU, and PH of Eggs Stored at 25 °C

The effects of different Se sources and storage time on weight loss, air chamber height, albumen height, HU, and pH in eggs stored at 25 °C are shown in Table 5. The weight loss, air chamber height, and albumen pH of eggs significantly increased, but the albumen height and HU significantly decreased from 0 to 6 wk (*p* < 0.001). Dietary Se supplementation had no influence on albumen pH (*p* > 0.05) but tended to reduce weight loss compared to the CON group in eggs stored at 25 °C (*p* = 0.056). Dietary SEC and SEC + SEY supplementation increased HU compared with the CON group in eggs stored at 25 °C (*p* < 0.05). There was a dietary treatment × storage time interaction observed for albumen pH (*p* < 0.05). The albumen pH in the SEC group was lower than that in the CON and SEC + SEY group in eggs stored at 25 °C for 4 wk (*p* < 0.05).

### 3.5. Yolk Color, Yolk Index, and Yolk PH of Eggs at 25 °C

As storage time increased, the yolk color and yolk pH of eggs significantly increased, whereas the yolk index decreased (*p* < 0.001, Table 6). The source of the Se used in dietary supplementation did not affect yolk color, yolk index and yolk pH (*p* > 0.05). There was a trend of dietary treatment × storage time interactions observed for the yolk index (*p* = 0.065). The yolk index of eggs that hens fed the diets supplemented with SS and SEC laid increased compared to the CON group in eggs stored at 25 °C for 6 wk (*p* < 0.05).

### 3.6. Albumen Foaming Capacity, Albumen Foam Stability, and Albumen Gel Firmness of Eggs at 25 °C

The albumen foaming capacity and gel firmness of eggs increased gradually with increased storage time (*p* < 0.001, Table 7). The source of the Se used in dietary supplementation had no influence on albumen foaming capacity and gel firmness of eggs storage at 25 °C (*p* > 0.05). However, dietary supplementation with Se tended to increase albumen foam stability compared to the CON group in eggs stored at 25 °C (*p* = 0.068). There was no interaction between dietary treatment and storage time on the albumen foaming capacity, foam stability, and gel firmness of eggs (*p* > 0.05).

### 3.7. Antioxidant Capacity of Egg Yolk at 4 °C and 25 °C

The T-AOC level and T-SOD activity of yolks decreased, but the yolk MDA content increased as the storage time lengthened (*p* < 0.001, Table 8). Dietary supplementation with SEC and SEC + SEY alleviated the decrease in yolk T-AOC level compared to eggs from the CON group (eggs stored at 4 °C and 25 °C) and SEY group (eggs stored at 25 °C) (*p* < 0.05). Moreover, dietary SS also increased the T-AOC level of yolks compared to the CON group in eggs stored at 25 °C (*p* < 0.05). The yolk T-SOD activity in the SS, SEY, and SEC groups was higher than that in the CON group (eggs stored at 4 °C and 25 °C) and the SEC + SEY group (eggs stored at 4 °C) (*p* < 0.001). Moreover, dietary Se supplementation decreased yolk MDA content compared to the CON group in eggs stored at 4 °C and 25 °C (*p* < 0.001). There was a dietary treatment × storage time interaction observed for yolk MDA and T-SOD in eggs stored at 4 °C (MDA, *p* < 0.05) and 25 °C (MDA, *p* < 0.001; T-SOD, *p* < 0.05), and a trend of dietary treatment × storage time interactions observed for yolk T-SOD activity (*p* = 0.079) in eggs stored at 4 °C. Dietary SEC, SEY, and SEC + SEY supplementation decreased the yolk MDA level in eggs stored for 0 wk (SEY and SEC + SEY, stored at 4 °C and 25 °C ), 2 wk (SEC and SEC + SEY stored at 4 °C and 25 °C), 4 wk (SEY, SEC, and SEC + SEY, stored at 25 °C), and 6 wk (SEC + SEY, stored at 25 °C). In the contrast, dietary SEC, SEY, and SEC + SEY supplementation increased yolk T-SOD activity in eggs stored for 0 wk (SEC, stored at 4 °C and 25 °C), 2 wk (SEY, stored at 4 °C), 4 wk, and 6 wk (SEC, SEY, and SEC + SEY, stored at 4 °C and 25 °C) (*p* < 0.05).

## 4. Discussion

*Cardamine violifolia*, as a natural Se hyperaccumulator, has been reported to demonstrate a variety of physiological effects, including anti-oxidation, anti-fatigue, weight loss and anti-aging in the brain [24,25]. As a natural antioxidant, SEC improved the egg quality and the ovarian antioxidant capacity of hens in the late phase [19]. Moreover, natural antioxidants of plant origin have been found to preserve egg quality during storage [11]. Therefore, *Cardamine violifolia* is expected to develop as new source of organic Se for animal production. 

Eggs are living things. After leaving the mother’s body, their quality gradually decreases, especially under the influence of temperature [15]. Weight loss is an important indicator in monitoring the freshness of an egg [26]. In the present study, weight loss and air chamber height increased as storage time increased at 4 °C and 25 °C, which is in agreement with previous studies [27,28]. The increase in weight loss of eggs during storage might be due to the continuous evaporation of water and loss of carbon dioxide through the pores of the eggs’ shells [29]. Consequently, as the volume of egg contents reduced, the air chamber height increased. In the present study, dietary Se supplementation tended to decrease weight loss. Consistent with our study, Nemati et al. [30] demonstrated that dietary supplementation with Se in laying hens alleviated the increase in weight loss of eggs during storage periods. 

In the current study, as the storage time increased, the yolk pH and yolk color increased, whereas the yolk index decreased during storage at 4 °C and 25 °C. During storage, water is constantly transferred from the albumen to the egg yolk through the egg yolk membrane. The additional water will stretch the yolk membrane, causing the yolk to flatten and the yolk index to decrease [15]. In the present study, dietary supplementation with Se from different sources had no effect on yolk pH or yolk color. However, dietary supplementation with SS and SEC tended to mitigate the decrease of the yolk index in eggs stored at 25 °C. Consistent with our findings, previous studies demonstrated that Se enrichment increased the yolk index during storage by enhancing the strength of the egg yolk membrane and reducing the transfer of albumen water to the yolk [15,31]. Our results indicated a positive effect of feeding laying hens a diet supplemented with SS or SEC on preserving the yolk quality during storage.

Albumen height and HU are important parameters of albumen quality and represent the freshness of an egg [4]. The storage of eggs alters their albumen quality and leads to a decrease in albumen height and HU. Our results indicated that albumen height and HU decreased during storage at 4 °C and 25 °C. During the storage period, the enzymes in the albumen hydrolyze the amino acid chains, leading to disruptions of the protein structure and the release of water. Consequently, the albumen is liquefied and the viscosity of the dense albumen decreases [32]. The liquefaction of the dense albumen causes a decrease in HU values [5]. However, previous researches reported that Se supplementation alleviated the decline in the HU of eggs during storage [2,33]. Similarly, we also found that dietary supplementation with SEC and SEC + SEY retarded the decrease in HU during storage of eggs at 4 °C and 25 °C. Our results suggested that SEC or SEC + SEY might be effective in preserving the albumen quality of eggs during storage.

Albumen pH is a useful indicator for evaluating changes in albumen quality, specifically as quality relates to albumen functionality in food. High quality egg albumen is desired for its foaming and gelling properties [34]. Albumen pH is positively correlated with albumen gel firmness and negatively correlated with albumen foaming stability [35,36]. Our results showed that the albumen pH increased with extended egg storage time at 4 °C and 25 °C. These results are in agreement with Adamski et al. [37] and Tabib et al. [38], who demonstrated that albumen pH increases during storage. The increased albumen pH could be due to carbon dioxide escaping through the eggshell pores during storage [39]. Moreover, we also found that SEC or SEC + SEY supplementation decreased albumen pH and albumen gel firmness. This suggests that SEC or SEC + SEY might act as a retarder of carbon dioxide diffusion, thereby SEC or SEC + SEY slows down an increase in albumen pH and gel firmness. Kirunda et al. [34] indicated that supplementation of a hen’s diet with vitamin E improved her eggs’ albumen foaming stability. Kirunda and Mckee [40] verified that albumen foaming stability was related to the effects of antioxidants. Moreover, previous studies have shown that natural antioxidant supplementation in the diets of laying hens can prolong the shelf life of egg albumens during storage [41,42]. Our results showed that dietary supplementation with SEC and SEC + SEY increased albumen foam stability. The enhanced albumen foam stability of SEC and SEC + SEY during storage might be due to the reduction of oxidative stress. These results indicated that SEC and SEC + SEY maintained albumen functionality during storage by retarding the increase in albumen pH and improving albumen foam stability. 

Nasri et al. [43] demonstrated that prolonged storage of eggs increased the oxidative stress on their yolks. In the present study, the T-AOC level and T-SOD activity of yolks decreased and the yolk MDA level increased as the storage time lengthened. As a metabolic product of lipid peroxidation, the level of MDA represents the attacking degree of free radicals. T-AOC reflects the total levels of non-enzymatic antioxidants. The T-SOD plays a crucial role in eliminating superoxide anion radicals. However, dietary Se supplementation decreased the yolk MDA content in eggs stored at 4 °C and 25 °C. Se plays an important role in maintaining redox balance and resisting oxidative stress. Se can indirectly induce the expression of antioxidant proteins such as SOD and catalase (CAT), thereby protecting organs and tissues against oxidative damage [44]. Dietary Se supplementation increased GSH-Px, T-SOD, and CAT activity in the serum and livers of hens [45]. Our study showed that SEC, SEY, or SEC + SEY alleviated the decrease in yolk T-AOC level and T-SOD activity in eggs stored at 4 °C and 25 °C. Similar results were obtained by Chen et al. [15], who reported that dietary supplementation with Se improved yolk antioxidant status during storage. In the present study, SEC and SEY were rich in organic Se. Compared with inorganic Se, organic Se has higher bioavailability. Our results indicated that SEC, SEY, or SEC + SEY enhanced the antioxidant capacity of egg yolks during storage by increasing the activity of antioxidant enzymes and inhibiting lipid peroxidation. It suggested that intervention to supply hens with dietary SEC, SEY or SEC + SEY might enhance the storage stability of egg yolks.

## 5. Conclusions

In conclusion, we demonstrated that dietary supplementation with Se-enriched *Cardamine violifolia* retarded the decrease in HU and the increase in albumen pH in stored eggs. Supplementation also improved albumen foam stability, thereby mitigating albumen quality loss during storage. *Cardamine violifolia* supplementation enhanced the antioxidant capacity of egg yolks and maintained yolk quality during storage. These findings suggest that dietary supplementation with SEC would be beneficial in extending the shelf life of eggs. Furthermore, this study might provide nutritional strategies to preserve egg quality during storage. *Cardamine violifolia* could be developed as a feed additive to extend the storage time of eggs.

## Figures and Tables

**Table 1:** Composition and nutrient level of basal diet for laying hens (air dry basis) ^1^.

Ingredients		Nutrients Levels ^3^	
Corn	63.25	Metabolizable energy (MJ/kg)	11.62
Soybean meal	25.45	Crude protein	16.31
Limestone	8.00	Calcium	3.66
Calcium hydrogen phosphate	1.50	Available phosphorus	0.41
Sodium chloride	0.30	Lysine	0.35
Premix ^2^	0.50	Selenium ^4^ (mg/kg)	0.156
DL-methionine	0.10		

^1^ Composition and nutrient level of basal diet for laying hens based on Wang et al. [19]. ^2^ The premix supplied the following (per kilogram of diet): vitamin A, 8000 IU; Vitamin D3, 3000 IU; Vitamin E, 20 IU; Vitamin K, 2.5 mg; cobalamine, 23 μg; pantothenate, 8 mg; folacin, 1 mg; Vitamin B1, 2.5 mg; Vitamin B2, 5.5 mg; niacin, 30 mg; Vitamin B6, 4 mg; Vitamin B12, 20 mg; biotin, 55 μg; choline chloride, 500 mg; iron, 90 mg; copper, 8 mg; zinc, 80 mg; manganese, 90 mg; iodine, 0.6 mg. ^3^ Nutrient levels were calculated values. ^4^ Se level is measured value.

**Table 2:** Effects of different Se sources and storage times on weight loss, air chamber height, albumen height, HU, and albumen pH of eggs stored at 4 °C ^1^.

Item	Storage Time (St) ^2^	Diet (D) ^3^					SEM	*p*-Value		
		CON	SS	SEY	SEC	SEC + SEY		D	St	D × St ^3^
Weight loss, %	0 wk	0.00	0.00	0.00	0.00	0.00	0.000	0.750	<0.001	0.998
	2 wk	2.24	2.12	2.23	2.18	2.21	0.059			
	4 wk	3.18	3.00	3.24	3.05	3.17	0.153			
	6 wk	5.15	5.07	5.21	5.23	5.19	0.209			
Air chamber height, cm	0 wk	0.22	0.19	0.22	0.18	0.19	0.015	0.441	<0.001	0.565
	2 wk	0.35	0.31	0.30	0.35	0.33	0.019			
	4 wk	0.39	0.37	0.37	0.39	0.38	0.023			
	6 wk	0.45	0.46	0.48	0.49	0.48	0.023			
Albumen height, mm	0 wk	5.81	6.28	6.16	6.13	6.18	0.273	0.032	<0.001	0.239
	2 wk	4.61	4.83	5.45	4.85	5.67	0.246			
	4 wk	4.32	5.53	4.82	5.25	5.31	0.294			
	6 wk	4.88	5.31	4.87	4.82	4.78	0.207			
HU	0 wk	72.38	79.26	76.07	77.27	77.43	1.835	0.015	<0.001	0.352
	2 wk	62.48	65.42	68.93	67.29	72.45	2.185			
	4 wk	59.05	68.39	63.92	69.14	69.75	2.690			
	6 wk	65.94	69.86	66.14	66.92	67.05	2.457			
Albumen pH	0 wk	8.61	8.65	8.67	8.77	8.73	0.045	0.225	<0.001	0.042
	2 wk	9.20	9.16	9.16	9.18	9.16	0.014			
	4 wk	9.22	9.33	9.30	9.29	9.26	0.028			
	6 wk	9.28	9.30	9.29	9.26	9.28	0.016			

^1^ Data are means of 6 replicates of 2 samples each. SEM, standard error of mean. CON, the diet supplemented with 0 mg/kg selenium (Se) (basal diet); SS, the diet supplemented with 0.3 mg/kg Se from sodium selenite; SEY, the diet supplemented with 0.3 mg/kg Se from Se-enriched yeast; SEC, the diet supplemented with 0.3 mg/kg Se from Se-enriched *Cardamine violifolia*; SEC + SEY, the diet supplemented with 0.3 mg/kg Se from Se-enriched *Cardamine violifolia* and 0.3 mg/kg Se from Se-enriched yeast; HU, Haugh unit. ^2^ St means storage time. ^3^ D means diet and D × St means the interaction between diet and storage time.

**Table 3:** Effects of different Se sources and storage times on yolk color, yolk index, and yolk pH of eggs stored at 4 °C ^1^.

Item	Storage Time (St) ^2^	Diet (D) ^3^					SEM	*p*-Value		
		CON	SS	SEY	SEC	SEC + SEY		D	St	D × St ^3^
Yolk color	0 wk	6.00	6.40	6.11	5.60	6.50	0.408	0.429	<0.001	0.154
	2 wk	6.90	6.50	6.70	7.50	6.50	0.390			
	4 wk	7.80	8.30	8.22	8.00	7.90	0.254			
	6 wk	7.10	8.30	7.44	7.80	8.40	0.296			
Yolk index	0 wk	0.37	0.39	0.36	0.37	0.37	0.012	0.144	<0.001	0.622
	2 wk	0.39	0.41	0.44	0.39	0.41	0.009			
	4 wk	0.39	0.43	0.39	0.39	0.41	0.018			
	6 wk	0.41	0.41	0.42	0.40	0.40	0.009			
Yolk pH	0 wk	6.17	6.32	6.25	6.25	6.19	0.057	0.365	<0.001	0.206
	2 wk	6.35	6.38	6.45	6.22	6.42	0.089			
	4 wk	6.80	6.90	6.78	6.59	6.79	0.132			
	6 wk	6.80	7.05	6.70	7.31	6.68	0.162			

^1^ Data are means of 6 replicates of 2 samples each. SEM, standard error of mean. CON, the diet supplemented with 0 mg/kg selenium (Se) (basal diet); SS, the diet supplemented with 0.3 mg/kg Se from sodium selenite; SEY, the diet supplemented with 0.3 mg/kg Se from Se-enriched yeast; SEC, the diet supplemented with 0.3 mg/kg Se from Se-enriched *Cardamine violifolia;* and SEC + SEY, the diet supplemented with 0.3 mg/kg Se from Se-enriched *Cardamine violifolia* and 0.3 mg/kg Se from Se-enriched yeast. ^2^ St means storage time. ^3^ D means diet and D × St means the interaction between diet and storage time.

**Table 4:** Effects of different Se sources and storage times on the albumen functional parameters of eggs stored at 4 °C ^1^.

Item	Storage Time (St) ^2^	Diet (D) ^3^					SEM	*p*-Value		
		CON	SS	SEY	SEC	SEC + SEY		D	St	D × St ^3^
Albumen foaming capacity, %	0 wk	352.00	354.67	365.33	358.67	349.33	8.320	0.271	<0.001	0.211
	2 wk	372.00	390.67	406.67	383.33	379.33	8.637			
	4 wk	348.70	364.00	352.67	374.67	362.67	11.055			
	6 wk	372.00	390.00	377.33	388.00	380.00	7.222			
Albumen foam stability, %	0 wk	73.48	77.33	74.96	74.81	75.99	1.161	0.145	<0.001	0.012
	2 wk	71.33	71.87	69.47	70.53	71.47	0.703			
	4 wk	67.92	71.20	73.60	75.67	75.53	1.587			
	6 wk	74.33	74.40	74.13	74.20	75.87	0.771			
Albumen gel firmness, N	0 wk	0.67	0.84	0.78	0.77	0.70	0.051	0.126	<0.001	0.002
	2 wk	0.97	1.05	0.94	0.86	0.95	0.061			
	4 wk	1.03	1.01	1.00	0.97	1.03	0.077			
	6 wk	1.30	1.02	1.01	1.19	0.85	0.070			

^1^ Data are means of 6 replicates of 2 samples each. SEM, standard error of mean. CON, the diet supplemented with 0 mg/kg selenium (Se) (basal diet); SS, the diet supplemented with 0.3 mg/kg Se from sodium selenite; SEY, the diet supplemented with 0.3 mg/kg Se from Se-enriched yeast; SEC, the diet supplemented with 0.3 mg/kg Se from Se-enriched *Cardamine violifolia*; and SEC + SEY, the diet supplemented with 0.3 mg/kg Se from Se-enriched *Cardamine violifolia* and 0.3 mg/kg Se from Se-enriched yeast. ^2^ St means storage time. ^3^ D means diet and D × St means the interaction between diet and storage time.

**Table 5:** Effects of different Se sources and storage times on weight loss, air chamber height, albumen height, HU, and albumen pH of eggs stored at 25 °C ^1^.

Item	Storage Time (St) ^2^	Diet (D) ^3^					SEM	*p*-Value		
		CON	SS	SEY	SEC	SEC + SEY		D	St	D × St ^3^
Weight loss, %	0 wk	0.00	0.00	0.00	0.00	0.00	0.000	0.054	<0.001	0.146
	2 wk	2.48	2.42	2.37	2.31	2.42	0.091			
	4 wk	5.96	5.77	5.40	5.63	5.81	0.304			
	6 wk	8.81	7.29	7.30	7.45	7.11	0.317			
Air chamber height, cm	0 wk	0.22	0.20	0.21	0.17	0.19	0.016	0.394	<0.001	0.485
	2 wk	0.38	0.35	0.38	0.39	0.35	0.025			
	4 wk	0.44	0.46	0.43	0.46	0.46	0.030			
	6 wk	0.61	0.48	0.51	0.54	0.52	0.032			
Albumen Height, mm	0 wk	6.06	6.17	5.65	6.06	6.47	0.224	0.333	<0.001	0.217
	2 wk	3.06	3.69	3.32	3.25	3.65	0.139			
	4 wk	3.26	3.15	3.30	3.42	3.10	0.136			
	6 wk	2.35	2.46	2.28	2.39	2.31	0.098			
HU	0 wk	73.66	75.53	71.67	74.68	76.92	1.844	0.014	<0.001	0.599
	2 wk	39.65	48.39	45.43	45.88	50.42	2.425			
	4 wk	40.27	42.53	43.23	48.52	48.58	2.021			
	6 wk	33.18	35.81	32.85	34.32	33.52	1.441			
Albumen pH	0 wk	8.61	8.65	8.67	8.63	8.73	0.045	0.202	<0.001	0.017
	2 wk	9.54	9.54	9.55	9.46	9.54	0.025			
	4 wk	9.68	9.60	9.64	9.50	9.72	0.071			
	6 wk	9.73	9.70	9.70	9.65	9.71	0.025			

^1^ Data are means of 6 replicates of 2 samples each. SEM, standard error of mean. CON, the diet supplemented with 0 mg/kg selenium (Se) (basal diet); SS, the diet supplemented with 0.3 mg/kg Se from sodium selenite; SEY, the diet supplemented with 0.3 mg/kg Se from Se-enriched yeast; SEC, the diet supplemented with 0.3 mg/kg Se from Se-enriched *Cardamine violifolia*; SEC + SEY, the diet supplemented with 0.3 mg/kg Se from Se-enriched *Cardamine violifolia* and 0.3 mg/kg Se from Se-enriched yeast; HU, Haugh unit. ^2^ St means storage time. ^3^ D means diet and D × St means the interaction between diet and storage time.

**Table 6:** Effects of different Se sources and storage times on yolk color, yolk index and yolk pH of eggs stored at 25 °C ^1^.

Item	Storage Time (St) ^2^	Diet (D) ^3^					SEM	*p*-Value		
		CON	SS	SEY	SEC	SEC + SEY		D	St	D × St ^3^
Yolk color	0 wk	6.67	6.00	7.00	5.50	6.50	0.408	0.939	<0.001	0.719
	2 wk	7.00	8.00	8.00	7.50	6.50	0.414			
	4 wk	9.00	8.50	8.00	8.25	9.00	0.164			
	6 wk	8.67	9.50	8.50	8.75	9.00	0.159			
Yolk index	0 wk	0.38	0.38	0.37	0.36	0.37	0.011	0.642	<0.001	0.065
	2 wk	0.26	0.30	0.29	0.28	0.30	0.010			
	4 wk	0.21	0.20	0.23	0.23	0.21	0.016			
	6 wk	0.12	0.16	0.14	0.16	0.15	0.016			
Yolk pH	0 wk	6.15	6.44	6.36	6.27	6.19	0.059	0.681	<0.001	0.165
	2 wk	6.41	6.44	6.72	6.68	6.74	0.132			
	4 wk	6.81	7.67	6.63	7.11	7.06	0.394			
	6 wk	7.57	7.20	7.77	7.19	7.23	0.217			

^1^ Data are means of 6 replicates of 2 samples each. SEM, standard error of mean. CON, the diet supplemented with 0 mg/kg selenium (Se) (basal diet); SS, the diet supplemented with 0.3 mg/kg Se from sodium selenite; SEY, the diet supplemented with 0.3 mg/kg Se from Se-enriched yeast; SEC, the diet supplemented with 0.3 mg/kg Se from Se-enriched *Cardamine violifolia*; and SEC + SEY, the diet supplemented with 0.3 mg/kg Se from Se-enriched *Cardamine violifolia* and 0.3 mg/kg Se from Se-enriched yeast. ^2^ St means storage time. ^3^ D means diet and D × St means the interaction between diet and storage time.

**Table 7:** Effects of different Se sources and storage time on the albumen functional parameters of eggs stored at 25 °C ^1^.

Item	Storage Time (St) ^2^	Diet (D) ^3^					SEM	*p*-Value		
		CON	SS	SEY	SEC	SEC + SEY		D	St	D × St ^3^
Albumen foaming capacity, %	0 wk	351.00	353.00	356.00	371.00	345.00	8.721	0.757	<0.001	0.306
	2 wk	358.67	385.19	371.11	376.67	363.00	10.663			
	4 wk	373.33	353.33	365.19	350.00	368.00	8.672			
	6 wk	370.67	379.26	388.52	373.33	390.67	6.678			
Albumen foam stability, %	0 wk	74.53	76.53	74.53	73.26	75.33	1.317	0.068	0.707	0.139
	2 wk	73.06	73.48	74.81	75.40	74.22	0.876			
	4 wk	70.38	71.92	75.04	75.58	78.00	1.516			
	6 wk	70.80	76.13	77.06	76.41	73.55	1.656			
Albumen gel firmness, N	0 wk	0.72	0.89	0.81	0.85	0.73	0.060	0.221	<0.001	0.508
	2 wk	0.83	0.75	0.69	0.82	0.80	0.055			
	4 wk	1.04	1.22	1.07	1.04	0.93	0.089			
	6 wk	1.48	1.52	1.46	1.50	1.32	0.091			

^1^ Data are means of 6 replicates of 2 samples each. SEM, standard error of mean. CON, the diet supplemented with 0 mg/kg selenium (Se) (basal diet); SS, the diet supplemented with 0.3 mg/kg Se from sodium selenite; SEY, the diet supplemented with 0.3 mg/kg Se from Se-enriched yeast; SEC, the diet supplemented with 0.3 mg/kg Se from Se-enriched *Cardamine violifolia*; and SEC + SEY, the diet supplemented with 0.3 mg/kg Se from Se-enriched *Cardamine violifolia* and 0.3 mg/kg Se from Se-enriched yeast. ^2^ St means storage time. ^3^ D means diet and D × St means the interaction between diet and storage time.

**Table 8:** Effects of different Se sources and storage times on yolk antioxidant capacity in eggs stored at 4 °C and 25 °C ^1^.

Item	Storage Time (St) ^2^	Diet (D) ^3^					SEM	*p*-Value		
		CON	SS	SEY	SEC	SEC + SEY		D	St	D × St ^3^
Stored at 4 °C										
T-AOC, mmol/g prot	0 wk	0.44	0.50	0.43	0.51	0.52	0.038	0.021	<0.001	0.396
	2 wk	0.18	0.19	0.23	0.24	0.25	0.017			
	4 wk	0.16	0.18	0.18	0.18	0.17	0.009			
	6 wk	0.02	0.02	0.03	0.05	0.07	0.003			
T-SOD, U/mg prot	0 wk	10.56	12.23	11.89	13.43	10.40	0.648	<0.001	<0.001	0.079
	2 wk	8.13	8.02	9.11	8.11	7.93	0.335			
	4 wk	5.34	6.64	7.10	6.47	6.15	0.277			
	6 wk	5.29	6.39	6.87	6.77	5.75	0.264			
MDA, nmol/mg prot	0 wk	1.17	0.92	0.74	1.03	0.68	0.034	<0.001	<0.001	0.041
	2 wk	1.30	1.10	1.12	1.05	1.00	0.036			
	4 wk	1.40	1.19	1.09	1.22	1.09	0.074			
	6 wk	1.38	1.31	1.30	1.17	1.10	0.086			
Stored at 25 °C										
T-AOC, mmol/g prot	0 wk	0.48	0.51	0.42	0.50	0.52	0.043	0.006	<0.001	0.251
	2 wk	0.14	0.16	0.15	0.15	0.16	0.009			
	4 wk	0.09	0.12	0.12	0.15	0.13	0.009			
	6 wk	0.02	0.02	0.03	0.06	0.07	0.003			
T-SOD, U/mg prot	0 wk	10.75	12.32	11.90	12.96	10.33	0.654	0.003	<0.001	0.017
	2 wk	6.54	7.35	7.72	7.21	6.66	0.426			
	4 wk	2.99	2.90	3.66	3.29	4.33	0.258			
	6 wk	3.02	3.73	3.77	4.88	3.72	0.261			
MDA, nmol/mg prot	0 wk	1.14	0.88	0.68	1.00	0.66	0.053	<0.001	<0.001	<0.001
	2 wk	1.31	0.92	1.03	0.92	0.98	0.032			
	4 wk	1.39	1.31	1.16	1.07	1.00	0.052			
	6 wk	1.41	1.41	1.32	1.15	1.07	0.087			

^1^ Data are means of 6 replicates of 2 samples each. SEM, standard error of mean. CON, the diet supplemented with 0 mg/kg selenium (Se) (basal diet); SS, the diet supplemented with 0.3 mg/kg Se from sodium selenite; SEY, the diet supplemented with 0.3 mg/kg Se from Se-enriched yeast; SEC, the diet supplemented with 0.3 mg/kg Se from Se-enriched *Cardamine violifolia*; SEC + SEY, the diet supplemented with 0.3 mg/kg Se from Se-enriched *Cardamine violifolia* and 0.3 mg/kg Se from Se-enriched yeast; T-AOC, total antioxidant capacity; T-SOD, total superoxide dismutase; MAD, malonaldehyde. ^2^ St means storage time. ^3^ D means diet and D × St means the interaction between diet and storage time.

## Data Availability

The original contributions presented in the study are included in the article, further inquiries can be directed to the corresponding author.
